# A new distinguishing attack on reduced round ChaCha permutation

**DOI:** 10.1038/s41598-023-39849-1

**Published:** 2023-08-26

**Authors:** Chandan Dey, Santanu Sarkar

**Affiliations:** Department of Mathematics, Indian Institute of Technology Madras, Chennai, India

**Keywords:** Computer science, Information technology

## Abstract

This work concentrates on differential-linear distinguishing attacks on the prominent ARX-based permutation ChaCha. Here, we significantly improve the 7-round differential-linear distinguisher for ChaCha permutation by introducing a new path of linear approximation. We first introduce a new single-bit differential distinguisher for the 3.5th round of the permutation that assists us in inventing a new path for the differential-linear distinguisher. We show that one can distinguish a 7-round ChaCha permutation with time complexity of $2^{207}$. This improves the recent work of Coutinho et al. (in: Advances in Cryptology—ASIACRYPT 2022—28nd International Conference on the Theory and Application of Cryptology and Information Security, Taipei, Taiwan, December 5–9, 2012, Springer, 2022), which achieved time complexity $2^{214}$. We also propose a distinguisher for the 7.25-round of ChaCha permutation and this is the first distinguishing attack for more than 7-round of ChaCha permutation. We provide theoretical proofs and the corresponding experimental results for the linear approximations that we use for differential-linear distinguisher. We point out that the existing multibit distinguishing attacks on the cipher ChaCha are invalid. These attacks are worked only for the ChaCha permutation.

## Introduction

In recent times, all necessary data needs to be encrypted; for this, reliable symmetric primitives play a significant role. There are many types of symmetric primitives. Among those, ARX-based symmetric primitives are one of the most famous categories because of their simple design and high performance in software, where ARX stands for Addition, Rotation, and XOR operations. Here the Addition is modular addition, i.e., for two *n* bits numbers, addition is performed modulo $2^n.$ Some famous ARX-based designs are as follows: block ciphers Speck^1^, Sparx^2^; stream ciphers Salsa20^3^, ChaCha^4^; MAC Chaskey^5^ and cryptographic permutation Sparkle^6^. The only nonlinear operation involved in ARX-based ciphers is modular addition. This attracts researchers to evaluate the differential and linear properties of the modular addition, and those properties are already analyzed in several works^7–9^. However, those analyses hold up to some reduced rounds of the ciphers, not for the full round.

ARX-based ciphers are well known not only for their simple design and efficiency but also for better security reasons. Since the modular addition operation is involved in the design of ARX-based ciphers, the algebraic degree attains nearly full degree after a few rounds. There is a way to create trust in these primitives by repeatedly analyzing their security and improving their security margin. The process of doing so is called cryptanalysis. Several cryptanalysis techniques have been proposed in the literature for the security evaluation of these primitives. Among those, differential cryptanalysis and linear cryptanalysis are the most famous statistical cryptanalysis methods. In this paper, we discuss the basic ideas of differential and linear attacks and the concept of combining these two attacks.

Both the primitives Salsa^3^ and ChaCha^4^ are renowned ARX-based stream ciphers designed by Bernstein. Both the ciphers have 20 rounds. In 2005, the 12-round version of Salsa was submitted by Bernstein to the eSTREAM project^10,11^ and later in 2007, it was selected as one of the finalists in the software category^10,11^. Bernstein proposed the stream cipher ChaCha, a variant of Salsa in 2008 with a few modifications in the round function. The diffusion speed in the round function of ChaCha is faster than Salsa, and it also has good resistance to attacks. That is why ChaCha is a prominent stream cipher, and it has several uses, e.g., Google adopted ChaCha to replace RC4 and ChaCha along with Poly1305^12^ is used in TLS^13^.

### Prior works

In 2005, Crowley^14^ first proposed a differential attack on 5 rounds of Salsa^3^, and this was the first third-party cryptanalysis on Salsa. In the following year, Fischer et al.^15^ improved the attack on Salsa by one round. At FSE 2008^16^, Aumasson et al. introduced the concept of probabilistic neutral bits (PNBs) for security analysis of the popular stream ciphers Salsa and ChaCha. In their work, they used differential attack and PNBs approach for key recovery of the reduced round version of Salsa and ChaCha. After that, Shi et al.^17^ proposed a column chaining distinguisher (CCD) for further enhancement of security analysis of the ciphers for both the versions, viz., 128 bits key and 256 bits key. In 2016, Maitra^18^ presented how to choose proper IVs to improve further the attack complexity for the reduced round version of both the ciphers. In the literature, Choudhuri et al.^19^ first proposed the multi-bit distinguisher for both the ciphers, where they extended the single-bit distinguisher for some rounds to multi-bit distinguisher for more rounds using linear approximations with the idea of differential-linear attack. Their work significantly improved the key recovery attack complexity for both the ciphers. Later, Dey et al.^20^ improved the attack complexity by modifying the set of PNBs, and also they presented the theoretical interpretation of their work in Ref.^21^. Another major improvement appeared at Crypto 2020, where Beierle et al.^22^ identified computationally the first single-bit distinguisher for the 3.5 rounds ChaCha, and Dey et al.^23^ proved this single-bit distinguisher theoretically. Also, using the obtained single-bit distinguisher, Beierle et al.^22^ improved the key recovery attack for 6 rounds ChaCha by partitioning technique and for 7 rounds ChaCha by PNBs approach. At Eurocrypt 2021^24^, Coutinho et al. proposed differential-linear distinguishers for 6 and 7 rounds ChaCha. Also, they provided some 3.5th round single-bit differential distinguishes, which were shown inaccurate by Dey et al.^23^. In the next Eurocrypt 2022, Dey et al.^25^ provided a modified algorithm to identify the better PNBs set and the algorithm is faster than the previous algorithm presented at^20^. This contribution significantly improved the key recovery attack complexity for 7 rounds ChaCha (256 bits key), and also they provided the first key recovery attack for 6.5 rounds ChaCha (128 bits key). Miyashita et al. introduced a first-time key recovery attack on 7.25 rounds of the cipher in^26^. According to Miyashita et al.^26^

The time complexity, data complexity, and success probability of the proposed attack are $2^{255.62}$, $2^{48.36}$, and 0.5, respectively. Although the proposed attack is less efficient than a brute force attack, it is the first dedicated attack on the target and provides both a baseline and useful components (i.e., differential bias and PNB) for improved attacks.

In our work, we present the first-time differential-linear distinguisher for 7.25 rounds of the permutation. Recently at Asiacrypt 2022^27^, Coutinho et al. improved the 7 rounds differential-linear distinguisher for ChaCha with data and time complexity $2^{214}$ though their obtained distinguisher is not valid for the cipher. We have improved this recent distinguisher complexity for the 7 rounds of the permutation.

### Our contribution

In this work, we introduce a new path of linear approximations from the 3.5th round to the 7th round, significantly improving the existing differential-linear distinguisher for the 7 rounds ChaCha permutation. We propose Lemma 4 that approximates two consecutive active bits in one round to less number of multiple active bits in the next round with the same probability as Lemma 7 of^24^. Because of this lemma, the number of active bits in the 7th round is controlled. Further, these fewer active bits in the 7th round help us extend the differential-linear distinguisher to the 7.25th round of the permutation. We list the comparison of our work with the previous improvements in the following Table 1. We explain the idea of massive data generation for the attack not mentioned in previous works^24,27^. The existing distinguishing attacks on 7-round ChaCha are not valid for the ChaCha cipher. These attacks are worked for ChaCha permutation only, and we point out this issue in this work.

**Table 1:** Comparison of attack complexities.

Rounds	Attack type	Time	Data	Memory	Works
7	Distinguisher	$2^{224}$	$2^{224}$	0	Eurocrypt 2021^24^
		$2^{214}$	$2^{214}$	0	Asiacrypt 2022^27^
		$2^{207}$	$2^{207}$	0	“Distinguisher for 7 rounds ChaCha permutation” section
7.25	Distinguisher	$2^{231}$	$2^{231}$	0	“Distinguisher for 7.25 rounds ChaCha permutation” section

### Paper organization

The paper is organized as follows. In “Introduction” section, we provide an introduction regarding related works and our work. In “Design of ChaCha” section, we present the design of the ARX-based stream cipher ChaCha. We review the concept of differential-linear attack scenario and the corresponding distinguisher complexity analysis in “Differential-linear attack scenario” section. “Attack scenario for ChaCha” section consists of the attack scenario for ChaCha. This section has four subsections. In “Differential distinguisher for the 3.5 rounds ChaCha” section, we provide the 3.5-round differential distinguisher. “Linear approximations for ChaCha” section consists of linear approximations among bits of different rounds of the cipher. We illustrate the 7 rounds differential-linear distinguisher in “Distinguisher for 7 rounds ChaCha permutation” section and the 7.25 rounds differential-linear distinguisher in “Distinguisher for 7.25 rounds ChaCha permutation” section. Finally, we conclude the paper in “Conclusion” section.

### Notations

Here, we introduce some notations that we use throughout the paper.

- $\boxplus$ denotes the addition modulo $2^{32}$, $\oplus$ denotes the XOR operation, $x\lll y$ denotes the left rotation of the word *x* by *y* bits.
- $S^r$ denotes the $4 \times 4$ state matrix after *r* rounds and $S_i^r$ for $i\in \{0,1,\ldots ,15\}$ are 32 bits entries (word) of the state matrix.
- $S_i^r[j]$ denotes the *j*th bit of the *i*th word of the state matrix after *r* rounds.
- By $S_i^r[j_1,j_2,\ldots ,j_n]$ we mean the XOR of the bits $S_i^r[j_1], S_i^r[j_2],\ldots ,S_i^r[j_n],$ i.e., $S_i^r[j_1,j_2,\ldots ,j_n]=S_i^r[j_1]\oplus S_i^r[j_2] \oplus \cdots \oplus S_i^r[j_n].$
- (*i*, *j*) denotes the *j*th bit of the *i*th word.
- For two state matrices *S* and $\widetilde{S}$, the differential state matrix is denoted by $\Delta S=S\oplus \widetilde{S}.$
- $|\Delta S|$ denotes the Hamming weight of the differential state matrix $\Delta S.$

## Design of ChaCha

In this section, we discuss the design of the well-known stream cipher ChaCha.

The state of ChaCha consists of 16 words that are represented in the form of a $4 \times 4$ matrix. In the state matrix of ChaCha, each word is of 32 bits, and the state size is 512 bits. The state matrix of ChaCha initializes with four constant words $c_0=0x61707865, c_1=0x3320646e, c_2=0x79622d32, c_3=0x6b206574$ in the first row, eight key words $k_0, k_1,\ldots , k_7$ in the second and third row and one counter $t_0$, three nonces $v_0,v_1,v_2$ in the fourth row. We treat the nonces and the counter as IVs. The initial state matrix $(S^{0})$ is given in the following.

$$\begin{aligned} S^{0} = \left( \begin{array}{cccc} S_0^{0} &{} S_1^{0} &{} S_2^{0} &{} S_{3}^{0}\\ S_4^{0} &{} S_5^{0} &{} S_6^{0} &{} S_{7}^{0}\\ S_8^{0} &{} S_9^{0} &{} S_{10}^{0} &{} S_{11}^{0}\\ S_{12}^{0} &{} S_{13}^{0} &{} S_{14}^{0} &{} S_{15}^{0} \end{array}\right) = \left( \begin{array}{cccc} c_0 &{} c_1 &{} c_2 &{} c_3\\ k_0 &{} k_1 &{} k_2 &{} k_{3}\\ k_4 &{} k_5 &{} k_{6} &{} k_{7}\\ t_0 &{} v_{0} &{} v_{1} &{} v_{2} \end{array}\right) . \end{aligned}$$

The state matrix of ChaCha is updated using the quarter-round function (QRF), which operates on a 4-tuple $(S_a^{r}, S_b^{r}, S_c^{r}, S_d^{r})$ in the following manner.

$$\begin{aligned}{}&S_a^{r+\frac{1}{2}} =S_a^{r}\boxplus S_b^{r},\\&S_d^{r+\frac{1}{2}}=\left( \left( S_d^{r}\oplus S_a^{r+\frac{1}{2}}\right) \lll 16\right) ,\\&S_c^{r+\frac{1}{2}} = S_c^{r}\boxplus S_d^{r+\frac{1}{2}}, \\ {}&S_b^{r+\frac{1}{2}}=\left( \left( S_b^{r}\oplus S_c^{r+\frac{1}{2}}\right) \lll 12\right) ,\\&S_a^{r+1} =S_a^{r+\frac{1}{2}}\boxplus S_b^{r+\frac{1}{2}}, \\ {}&S_d^{r+1}=\left( \left( S_d^{r+\frac{1}{2}}\oplus S_a^{r+1}\right) \lll 8\right) ,\\&S_c^{r+1} =S_c^{r+\frac{1}{2}}\boxplus S_d^{r+1}, \\&S_b^{r+1}=\left( \left( S_b^{r+\frac{1}{2}}\oplus S_c^{r+1}\right) \lll 7\right) , \end{aligned}$$

i.e.,

$$\begin{aligned} \texttt{QRF} (S_a^{r},S_b^{r},S_c^{r},S_d^{r})\rightarrow (S_a^{r+1},S_b^{r+1},S_c^{r+1},S_d^{r+1}). \end{aligned}$$

**Figure 1:**
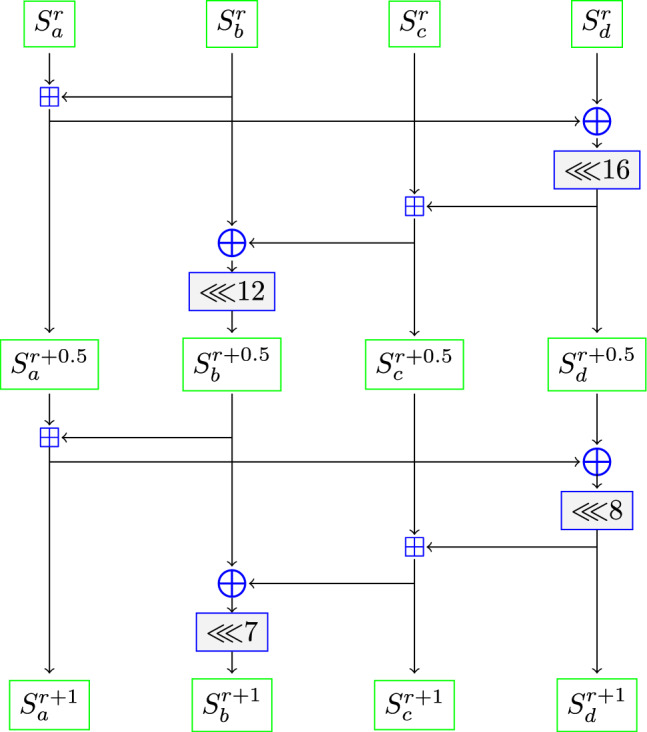
Diagram of quarter round function (QRF) applied on $(S_a^r,S_b^r,S_c^r,S_d^r)$.

The diagram of the quarter-round function (QRF) is presented in Fig. 1.

The round function of ChaCha constitutes four simultaneous applications of the quarter-round function ($\texttt{QRF}$). The $\texttt{QRF}$ operates on the four words of each column of the state matrix in odd rounds, and it operates on the four words of each diagonal of the state matrix in even rounds to update the state of the cipher. So, in the odd round or column round, the $\texttt{QRF}$ applies on $(S_a, S_b, S_c, S_d)$, where $(a,b,c,d)\in \{(0,4,8,12),(1,5,9,13),(2,6,10,14),(3,7,11,15)\}$ and in the even round or diagonal round, $\texttt{QRF}$ applies on $(S_a, S_b, S_c, S_d)$, where $(a,b,c,d)\in \{(0,5,10,15),(1,6,11,12),(2,7,8,13),$ $(3,4,9,14)\}$ to update the state matrix of the cipher.

In this work, in the $\texttt{QRF}$ operation, the update of $(S_a^{r}, S_b^{r}, S_c^{r}, S_d^{r})$ to $(S_a^{r+\frac{1}{2}}, S_b^{r}, S_c^{r}, S_d^{r+\frac{1}{2}})$ of the state matrix, we call this 0.25 round update of the cipher that we discuss in details at “Distinguisher for 7.25 rounds ChaCha permutation” section. The state matrix of ChaCha after *r* rounds is denoted by $S^{r}$, which is given in the following.

$$\begin{aligned} S^{r} = \left( \begin{array}{cccc} S_0^{r} &{} S_1^{r} &{} S_2^{r} &{} S_{3}^{r}\\ S_4^{r} &{} S_5^{r} &{} S_6^{r} &{} S_{7}^{r}\\ S_8^{r} &{} S_9^{r} &{} S_{10}^{r} &{} S_{11}^{r}\\ S_{12}^{r} &{} S_{13}^{r} &{} S_{14}^{r} &{} S_{15}^{r} \end{array}\right) . \end{aligned}$$

The key stream of the steam cipher ChaCha is generated as $Z=S^{0}\boxplus S^{R}$, where $S^{R}$ denotes the updated state of the cipher after *R*-round and output of the *R*-round ChaCha permutation. From the QRF of the cipher, it is clear that if one knows the 4-tuple $(S_a^{r+1}, S_b^{r+1}, S_c^{r+1}, S_d^{r+1})$ then he/she can find the value of the 4-tuple $(S_a^{r}, S_b^{r}, S_c^{r}, S_d^{r}).$ So, the QRF is reversible, and the round function of the cipher is reversible. For a more explicit discussion on the structure of ChaCha, we refer to^4^.

## Differential-linear attack scenario

In this section, we review the two significant cryptanalysis techniques and their consequences. The two main statistical cryptanalysis techniques for symmetric key primitives are differential cryptanalysis and linear cryptanalysis.

At Crypto 1990^28^, Biham and Shamir proposed the idea of differential cryptanalysis for security analysis of DES-like ciphers. Later this concept was used for security analysis of various types of symmetric key ciphers. In a differential attack, the attacker’s target is to identify an input difference that produces a fixed output difference with high probability. For that, the attacker has to generate many plaintexts with a fixed difference and then check using encryption oracle whether it returns ciphertexts with a fixed difference with high probability or not. Suppose one tracks the relationship between differences in input and differences in the corresponding output with a high probability for *r* rounds of the cipher; then we say that the cipher is distinguishable up to *r* rounds. Further, this high probability distinguisher helps to recover the secret key.

In 1993, Matsui^29^ introduced the notion of linear cryptanalysis and applied this technique to attack DES block cipher. The main idea of this attack is to find linear approximations across the rounds of the cipher that hold with high probability. Then the attacker tries to obtain the secret key using known plaintext ciphertext pairs and the linear approximation with a high probability.

In the following, we discuss how to combine differential attack and linear attack for further improvement of the attack.

### Differential-linear attack

In the paper^30^, Langford and Hellman proposed the idea of differential-linear attack. Here we discuss their idea of differential-linear attack. In their setting of differential-linear attack, the cipher *E* is divided into two parts, $E_1$ and $E_2$, i.e., $E=E_2\circ E_1$. The first part, $E_1$, corresponds to the differential distinguisher and the second part, $E_2$, corresponds to the linear distinguisher. Suppose $E_1$ consists of $r_1$ rounds of the cipher where $r_1$ rounds differential distinguisher is constructed and the next part $E_2$ consists of $r_2$ rounds where $r_2$ rounds linear distinguisher is constructed. The total $r_1+r_2$ rounds of differential-linear distinguishers are provided by the combination of both distinguishers.

At Crypto 2020^22^, Beirele et al. modified the classical setting of differential-linear attack by dividing the cipher into three parts: $E=E_2\circ E_m \circ E_1$, where the first part $(E_1)$ and middle part $(E_m)$ correspond to differential attack and the last part $(E_2)$ correspond to linear attack. Here $E_1$ and $E_m$ consist of $r_1$ and $r_m$ rounds, respectively, of the cipher where $r_1+r_m$ rounds differential distinguisher is constructed and the next part $E_2$ consists of $r_2$ rounds where $r_2$ rounds linear distinguisher is constructed. The combination of these distinguishers provides the total $r_1+r_m+r_2$ rounds differential-linear distinguisher.

#### Complexity analysis for distinguisher

Here, we explain how to calculate the complexity of the differential-linear attack. We start with two initial states of the cipher $S^0$ and $\widetilde{S}^0$, where $\widetilde{S}^0=S^0\oplus \Delta S^0$ and $\Delta S^0$ is the input difference. Then for the two-state matrices $S^r$ and $\widetilde{S}^r$ after the *r* rounds of the cipher, we consider the differential state matrix as $\Delta S^r=S^r\oplus \widetilde{S}^r$. The entries of the differential state matrix $\Delta S^r$ are denoted as $\Delta S^r_i$, where $\Delta S_i^r=S_i^r\oplus \widetilde{S}_i^r$ and $S_i^r$, $\widetilde{S}_i^r$ are the entries of the state matrices $S^r$ and $\widetilde{S}^r$ respectively. Suppose we consider two bits, i.e., the *j*th bits $S_i^r[j]$ and $\widetilde{S}_i^r[j]$ of the words $S_i^r$ and $\widetilde{S}_i^r$ respectively. Then the differential of the *j*th bit is defined by $\Delta S_i^r[j]=S_i^r[j]\oplus \widetilde{S}_i^r[j].$ We denote the linear combination of the bits of states $S_i^r$ and $\widetilde{S}_i^r$ as $\sigma$ and $\widetilde{\sigma }$ respectively, where $\sigma =\bigoplus _{i,j}S^{r}_i[j]$ and $\widetilde{\sigma }=\bigoplus _{i,j}\widetilde{S}^{r}_i[j].$ The linear combination of the differential of the bits is defined by $\Delta \sigma =\sigma \oplus \widetilde{\sigma }=\bigoplus _{i,j}\Delta S_i^r[j].$ For input difference $\Delta S^0$, let the probability of the *r* rounds differential distinguisher with the differential correlation $\epsilon _d$ is

$$\begin{aligned} \Pr(\Delta \sigma =0 \ | \ \Delta S^{0})=\frac{1}{2}(1+\epsilon _d). \end{aligned}$$

**Figure 2:**
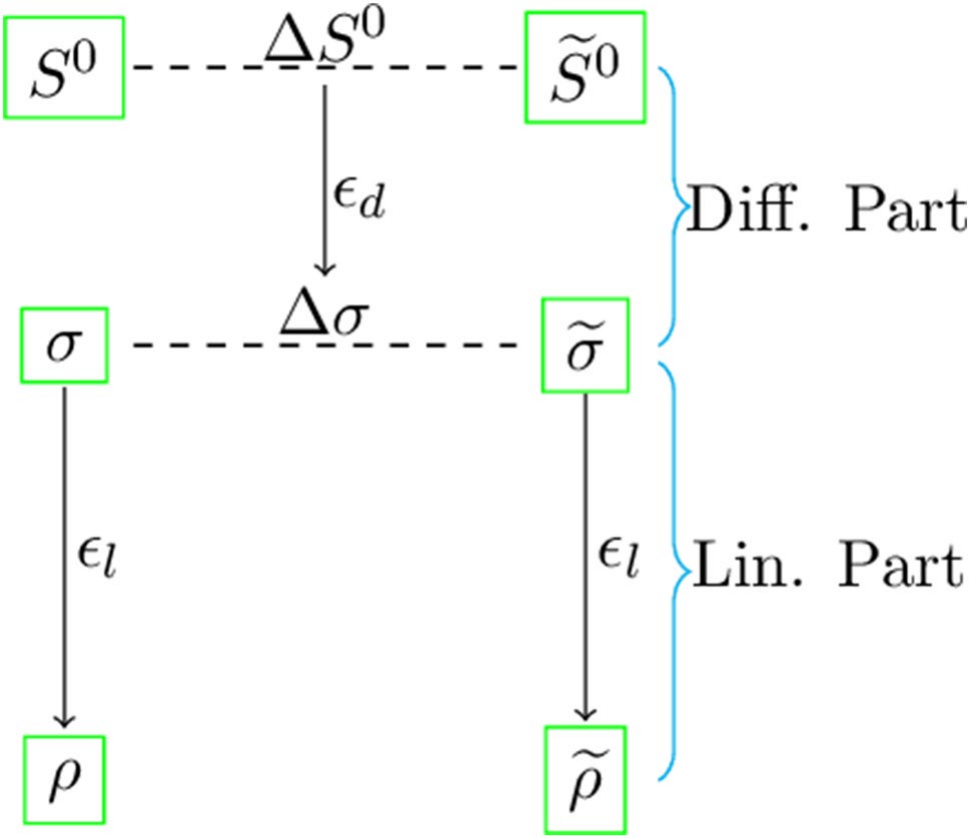
Differential-linear attack framework.

Now we extend this *r* rounds differential distinguisher to a few more rounds, i.e., $R(>r)$ rounds using the idea of linear cryptanalysis. For this, we find linear approximations between the states $S^r$ and $S^R$ of the cipher, and the same linear approximation holds between the states $\widetilde{S}^r$ and $\widetilde{S}^R$. Let the linear combination of the bits of the states $S^R$ and $\widetilde{S}^R$ are denoted by $\rho$ and $\widetilde{\rho }$ respectively, where $\rho =\bigoplus _{i,j}S_i^R[j]$ and $\widetilde{\rho }=\bigoplus _{i,j}\widetilde{S}_i^R[j].$ Then one can compute $\Delta \rho =\rho \oplus \widetilde{\rho }=\bigoplus _{i,j}\Delta S_i^R[j]$ similarly as above. Suppose the linear approximation between the states $S^r$ and $S^R$ holds with probability $\frac{1}{2}(1+\epsilon _{l})$, i.e., $\Pr(\sigma =\rho )=\frac{1}{2}(1+\epsilon _{l}),$ where $\epsilon _{l}$ is the linear correlation, and also this same linear approximation holds between the states $\widetilde{S}^r$ and $\widetilde{S}^R$. The differential-linear attack scenario is shown in Fig. 2. We aim to compute the differential-linear correlation of the *R* round differential-linear attack. We denote the differential-linear correlation by $\epsilon .$ For this, we have to find the probability of the event $\Delta \rho =0$ with the given input difference $\Delta S^0.$ So, for the differential-linear correlation $\epsilon$, the probability of the event $\Delta \rho =0$ is $\Pr(\Delta \rho =0)=\frac{1}{2}(1+\epsilon ).$ In the following, we compute the correlation $\epsilon$ in terms of differential correlation $\epsilon _d$ and linear correlation $\epsilon _l.$

Now,

$$\begin{aligned} \Pr(\Delta \sigma =\Delta \rho )&=\Pr(\sigma \oplus \widetilde{\sigma }=\rho \oplus \widetilde{\rho })\\&=\Pr(\sigma =\rho )\cdot \Pr(\widetilde{\sigma }=\widetilde{\rho })+\Pr(\sigma =\rho \oplus 1)\cdot \Pr(\widetilde{\sigma }=\widetilde{\rho }\oplus 1)\\&=\frac{1}{2}(1+\epsilon _l)\cdot \frac{1}{2}(1+\epsilon _l)+\frac{1}{2}(1-\epsilon _l)\cdot \frac{1}{2}(1-\epsilon _l)\\&=\frac{1}{2}(1+\epsilon _l^2). \end{aligned}$$

Then,

$$\begin{aligned} \Pr(\Delta \rho =0)&=\Pr(\Delta \sigma =0)\cdot \Pr(\Delta \sigma =\Delta \rho )+ \Pr(\Delta \sigma =1)\cdot \Pr\left( \Delta \sigma =\Delta \rho \oplus 1\right) \\&=\frac{1}{2}(1+\epsilon _d)\cdot \frac{1}{2}(1+\epsilon _l^2)+\frac{1}{2}(1-\epsilon _d)\cdot \frac{1}{2}(1-\epsilon _l^2)\\&=\frac{1}{2}(1+\epsilon _d \epsilon _l^2). \end{aligned}$$

Therefore, the required differential-linear correlation is $\epsilon =\epsilon _d \epsilon _l^2$ and the corresponding distinguisher complexity is $\mathcal {O}(\frac{1}{\epsilon _d^2 \epsilon _l^4})$. For a more detailed explanation of the differential-linear attack, we refer to^31^. Also, from the paper^32^, we know that for distinguishing between two events, where one event happens with probability *p*, and the other happens with probability $p(1+q),$ where *q* is small, we need $\mathcal {O}(\frac{1}{pq^2})$ random samples that give the constant probability of success.

## Attack scenario for ChaCha

In this section, we discuss the differential-linear attack framework for the cipher. As discussed in the previous section, here we divide the cipher into three subparts $E_1$, $E_m$, and $E_2$, where $E_1$ consists of the first round, $E_m$ consists of the next 2.5 rounds, and $E_2$ consists of the last linear part of the cipher. Here the targeted rounds for differential-linear attacks are 7 and 7.25 rounds of the ChaCha permutation.

### Differential distinguisher for the 3.5 rounds ChaCha

At Crypto 2020^22^, Beierle et al. obtained some 3.5 rounds of single-bit differential distinguishers experimentally. They chose input differences at (*p*, 6) for $p \in \{12,13,14,15\}$ and found differential correlation of $2^{-8.3}\approx 0.00317$ at (*i*, 0) for $i\in \{1,2,3,0\}$ respectively. In their attack, they minimized the Hamming weight of the differential state matrix after one round, i.e., after the $E_1$ subpart of the cipher in search of a better correlation at targeted rounds. They considered the Hamming weight 10 after one round, i.e., $|\Delta S^1|=10$ that holds with probability $2^{-5}$ on average, and for a detailed discussion on it, we refer to^22^. In the following, we highlight some of our observations regarding differential correlation for various input-output difference positions. For these observations, we have also minimized the Hamming weight $(|\Delta S^1|=10)$ of the differential state matrix after one round.

#### Observation 1

The input difference at position (*p*, 6) for $p\in \{12,13,14,15\}$ of the initial state of ChaCha result differential correlation 0.000002762 at output difference position (*q*, 0) for $q\in \{15,12,13,14\}$ of 3.5 rounds of ChaCha. We have observed these correlations using $2^{46}$ random samples.

#### Observation 2

The input difference at position (*p*, 6) for $p\in \{12,13,14,15\}$ of the initial state of ChaCha result differential correlation 0.00086 at output difference position (*q*, 0) for $q\in \{10,11,8,9\}$ of 3 rounds of ChaCha. We have observed these correlations using $2^{46}$ random samples.

**Figure 3:**
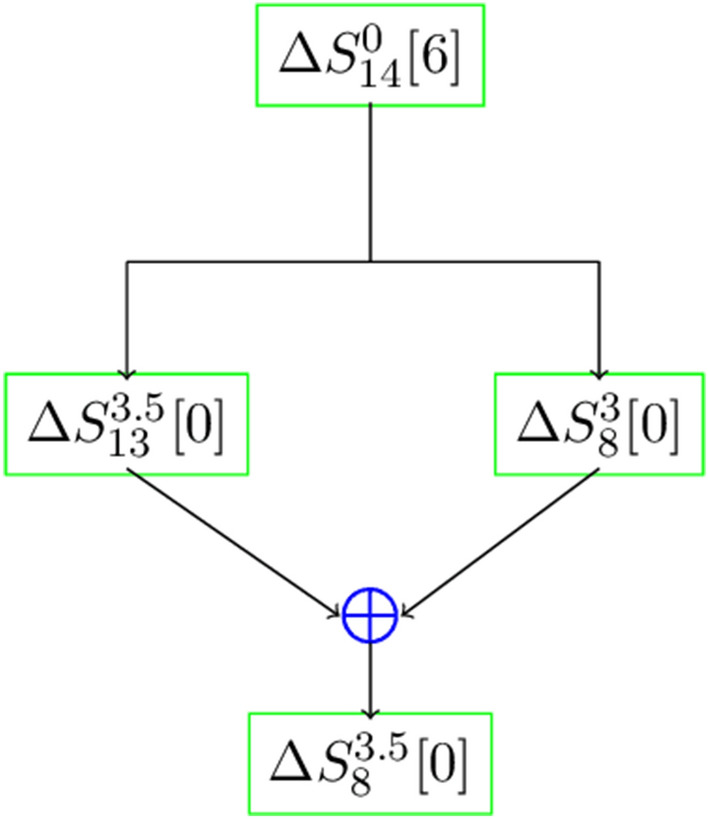
Differential distinguisher for the 3.5 rounds.

The above observations hold because of the symmetry in the round update for the state of ChaCha.

**Differential distinguisher:** Using input difference at (14, 6) in the initial state of ChaCha, we have experimentally obtained the forward differential correlation 0.000002762 at the output difference position (13, 0) for 3.5 rounds ChaCha. From the QRF, we get

$$\begin{aligned} S_c^{3.5}[0]=S_c^{3}[0]\oplus S_d^{3.5}[0]. \end{aligned}$$

Then for the diagonal indices $(a,b,c,d)=(2,7,8,13)$ the above relation becomes

$$\begin{aligned} S_8^{3.5}[0]=S_8^{3}[0]\oplus S_{13}^{3.5}[0]. \end{aligned}$$

Using the same input difference at (14, 6), we get the 3 rounds differential correlation 0.00086 at the output difference position (8, 0). In the state update of ChaCha, the quarter round function (QRF) operates column-wise i.e., $$(S_0, S_4, S_8, S_{12})$$, ($$S_1, S_5, S_9, S_{13})$$, $$(S_2, S_6, S_{10}, S_{14})$$, $$(S_3, S_7, S_{11}, S_{15})$$ respectively in the 3rd round and diagonally i.e., $$(S_0, S_5, S_{10}, S_{15})$$, $$(S_1, S_6, S_{11}, S_{12})$$, $$(S_2, S_7, S_8, S_{13})$$, $$(S_3, S_4, S_9, S_{14})$$ respectively in the 4th round. Since $$S_8$$ and $$S_{13}$$ are updated separately in the 3rd round and also in the update of $$S^{3.5}_{13}$$, it does not involve $$S^3_8$$, so $$S^3_8[0]$$ may not influence the bit $$S^{3.5}_{13}[0]$$. Therefore, using the Pilling-up Lemma^29^ as the two events are independent, the differential correlation at the position (8, 0) for 3.5 rounds ChaCha becomes $$\epsilon _d=0.000002762\times 0.00086\approx 2^{-28.65}.$$ This is presented in Fig. 3.

Similarly, from Observation 1 and Observation 2, we can derive the 3.5 rounds differential distinguishers with the same differential correlation as above for other pairs of input-output difference positions.

### Linear approximations for ChaCha

For ARX-based ciphers, the only nonlinear operation is the addition operation. In the addition of two 32 bits numbers *x* and *y*, the carry function is calculated as $\mathrm{Car}(x,y) = x\oplus y \oplus (x\boxplus y)$ and Car(x,y)[i] denotes the *i*th carry bit and also for the 0th bit $\mathrm{Car}(x,y)[0]=0.$ At Eurocrypt 2021^24^, Coutinho and Souza introduced the following linear approximations:

- 1 $$\begin{aligned} \begin{aligned}\Pr(\mathrm{Car}(x,y)[i]=y[i-1])=\frac{1}{2}\left( 1+\frac{1}{2}\right) \,\,\hbox {for}\,\,i>0. \end{aligned} \end{aligned}$$
- 2 $$\begin{aligned} \begin{aligned} \Pr(\mathrm{Car}(x,y)[i]\oplus \mathrm{Car}(x,y)[i-1]=0)=\frac{1}{2}\left( 1+\frac{1}{2}\right) \,\,\hbox {for}\,\,i>0. \end{aligned} \end{aligned}$$

According to Coutinho and Souza, these two probabilistic conditions are very useful for constructing linear approximations for more rounds of ARX-based ciphers. Here, we recall the work described in Refs.^19,24^. In those papers, the authors proposed many results to find linear approximations. In the following, we concentrate on some of their results that we will use to derive the linear approximations throughout this paper. We introduce a new path of differential-linear distinguisher using the following proposed lemmas.

#### Lemma 1

^19,24^ For one active input bit in round *r* and multiple active output bits in round $r+1$, the following linear approximations hold for $i>0$.

1. $$\begin{aligned} \begin{aligned} S_a^{r}[i]&=S_a^{r+1}[i]\oplus S_b^{r+1}[i+6,i+7,i+18,i+19] \oplus S_c^{r+1}[i+11,i+12]\\&\oplus S_d^{r+1}[i-1,i]\,\hbox {holds with probability}\, \frac{1}{2} \left( 1+\frac{1}{2^3}\right). \end{aligned} \end{aligned}$$
2. $$\begin{aligned} \begin{aligned} S_b^{r}[i]&=S_b^{r+1}[i+19]\oplus S_c^{r+1}[i,i+12] \oplus S_d^{r+1}[i-1,i]\\&\quad \hbox {holds with probability}\,\frac{1}{2}\left( 1+\frac{1}{2}\right) . \end{aligned} \end{aligned}$$
3. $$\begin{aligned} \begin{aligned} S_c^{r}[i]&=S_a^{r+1}[i-1,i]\oplus S_c^{r+1}[i] \oplus S_d^{r+1}[i-1,i,i+7,i+8]\\&\quad \hbox {holds with probability}\, \frac{1}{2} \left( 1+\frac{1}{2^2}\right) . \end{aligned} \end{aligned}$$
4. $$\begin{aligned} \begin{aligned} S_d^{r}[i]&=S_a^{r+1}[i,i+16]\oplus S_b^{r+1}[i+6,i+7]\oplus S_c^{r+1}[i-1,i]\oplus S_d^{r+1}[i+24]\\&\quad \hbox {holds with probability}\, \frac{1}{2} \left( 1+\frac{1}{2}\right) . \end{aligned} \end{aligned}$$

#### Proof

For proof, we refer to^19,24^. Q.E.D.

Here we rewrite Lemma 7 of^27^ into two different following lemmas with modified notations for the flow of our work.

#### Lemma 2

For two consecutive active bits in round *r* and multiple active output bits in $r+\frac{1}{2}$ rounds in the following linear approximations holds with probability $\frac{1}{2}(1+\frac{1}{2})$ for $i>0.$

$$\begin{aligned}{}&S_a^r[i]\oplus S_a^r[i-1]= S_a^{r+\frac{1}{2}}[i]\oplus S_b^{r+\frac{1}{2}}[i+12]\oplus S_c^{r+\frac{1}{2}}[i]\\&S_c^r[i]\oplus S_c^r[i-1]=S_c^{r+\frac{1}{2}}[i]\oplus S_d^{r+\frac{1}{2}}[i]. \end{aligned}$$

#### Proof

We refer to Lemma 7 of^27^ for the proof. Q.E.D.

#### Lemma 3

For two consecutive active bits in round $r+\frac{1}{2}$ and multiple active output bits in $r+1$ rounds in the following linear approximations holds with probability $\frac{1}{2}(1+\frac{1}{2})$ for $i>0.$

$$\begin{aligned}{}&S_a^{r+\frac{1}{2}}[i]\oplus S_a^{r+\frac{1}{2}}[i-1]= S_a^{r+1}[i]\oplus S_b^{r+1}[i+7]\oplus S_c^{r+1}[i]\\&S_c^{r+\frac{1}{2}}[i]\oplus S_c^{r+\frac{1}{2}}[i-1]=S_c^{r+1}[i]\oplus S_d^{r+1}[i]. \end{aligned}$$

#### Proof

We refer to Lemma 7 of^27^ for the proof. Q.E.D.

In an earlier analysis of linear approximations (Lemma 1), there were probabilistic linear approximations for a single bit active of one round with multiple active bits of the next round. The Lemma 7 of the paper^24^ presents the linear approximation between two consecutive active bits of one round and multiple active bits of the next round. Later, at Asiacrypt 2022^27^, Coutinho et al. proposed linear approximations (Lemmas 2, 3) between two consecutive active bits of one round and multiple active bits of the next half round. In the following, we present a lemma that forms a bridge between two consecutive active bits of one round and multiple active output bits of the next round with the same probability as Lemma 7 of^24^ but less number of active bits in the next round.

#### Lemma 4

The following linear approximations between two consecutive active bits in *r*th round and multiple active bits in $r+1$th round hold probabilistically for $i>0.$

1. $$\begin{aligned} \begin{aligned}{}&S_a^r[i-1,i]=S_a^{r+1}[i]\oplus S_b^{r+1}[i+6,i+7,i+19]\oplus S_c^{r+1}[i-1,i+12]\oplus S_d^{r+1}[i-1,i]\\&\quad \hbox {holds with probability}\,\frac{1}{2}\left( 1+\frac{1}{2^3}\right) . \end{aligned} \end{aligned}$$
2. $$\begin{aligned} \begin{aligned}{}&S_b^r[i-1,i]= S_b^{r+1}[i+18,i+19] \oplus S_c^{r+1}[i,i+11,i+12]\oplus S_d^{r+1}[i]\\&\quad \hbox {holds with probability}\,\frac{1}{2}\left( 1+\frac{1}{2}\right) . \end{aligned} \end{aligned}$$
3. $$\begin{aligned} \begin{aligned}{}&S_c^r[i-1,i]= S_a^{r+1}[i]\oplus S_c^{r+1}[i] \oplus S_d^{r+1}[i-1,i,i+8]\\&\quad \hbox {holds with probability}\,\frac{1}{2}\left( 1+\frac{1}{2^2}\right) . \end{aligned} \end{aligned}$$
4. $$\begin{aligned} \begin{aligned}{}&S_d^r[i-1,i]= S_a^{r+1}[i,i+15,i+16] \oplus S_b^{r+1}[i+7]\oplus S_c^{r+1}[i]\oplus S_d^{r+1}[i+23,i+24] \\&\quad \hbox {holds with probability}\,\frac{1}{2}\left( 1+\frac{1}{2}\right) . \end{aligned} \end{aligned}$$

#### Proof

For proving the lemma, we use the QRF and previous lemmas. The proofs are as follows:

1. $$\begin{aligned} \begin{aligned} S_a^r[i-1,i]=&~S_a^{r+\frac{1}{2}}[i]\oplus S_b^{r+\frac{1}{2}}[i+12]\oplus S_c^{r+\frac{1}{2}}[i]\,~\hbox{holds with probability}\,\frac{1}{2}\left( 1+\frac{1}{2}\right) \\&\quad (\hbox {Using}\, \hbox {Lemma}\,2)\\ =&~S_a^{r+1}[i]\oplus S_b^{r+\frac{1}{2}}[i-1,i]\oplus S_c^{r+1}[i+12]\oplus S_b^{r+1}[i+19] \oplus S_c^{r+1}[i]\\&\oplus S_d^{r+1}[i-1,i]\,~\hbox{holds with probability}\,\frac{1}{2}\left( 1+\frac{1}{2^3}\right) \\&\quad (\hbox {From}\,~\texttt{QRF} \,~\hbox{and using}\,~\hbox {Equation}\,1)\\ =&~S_a^{r+1}[i]\oplus S_c^{r+1}[i-1,i]\oplus S_b^{r+1}[i+6,i+7]\oplus S_c^{r+1}[i+12]\\&\oplus S_b^{r+1}[i+19]\oplus S_c^{r+1}[i]\oplus S_d^{r+1}[i-1,i]\\&\,\hbox {holds with probability}\,\frac{1}{2}\left( 1+\frac{1}{2^3}\right) \\ =&~S_a^{r+1}[i]\oplus S_b^{r+1}[i+6,i+7,i+19]\oplus S_c^{r+1}[i-1,i+12]\oplus S_d^{r+1}[i-1,i]\\&\quad \hbox {holds with probability}\,\frac{1}{2}\left( 1+\frac{1}{2^3}\right) . \end{aligned} \end{aligned}$$
2. $$\begin{aligned} \begin{aligned} S_b^r[i-1,i]&=S_c^{r+\frac{1}{2}}[i-1,i]\oplus S_b^{r+\frac{1}{2}}[i+11,i+12]\,\,(\hbox {From}\,~\texttt{QRF})\\&=S_c^{r+1}[i]\oplus S_d^{r+1}[i]\oplus S_c^{r+1}[i+11,i+12] \oplus S_b^{r+1}[i+18,i+19]\\&\quad \hbox {holds with probability}\,\frac{1}{2}\left( 1+\frac{1}{2}\right) \,(\hbox {From}\,~\texttt{QRF} \,~\hbox{and using}\,~\hbox{Lemma}\,3)\\&=S_b^{r+1}[i+18,i+19]\oplus S_c^{r+1}[i,i+11,i+12]\oplus S_d^{r+1}[i]\\&\quad \hbox {holds with probability}\,\frac{1}{2}\left( 1+\frac{1}{2}\right) . \end{aligned} \end{aligned}$$
3. $$\begin{aligned} \begin{aligned} S_c^r[i-1,i]&=S_c^{r+\frac{1}{2}}[i]\oplus S_d^{r+\frac{1}{2}}[i]\,~\hbox{holds with probability}\,\frac{1}{2}\left( 1+\frac{1}{2}\right) \,\,(\hbox {Using}\,~\hbox{Lemma}\,2)\\&=S_c^{r+1}[i]\oplus S_d^{r+1}[i-1,i]\oplus S_a^{r+1}[i]\oplus S_d^{r+1}[i+8]\\&\quad \hbox {holds with probability}\,\frac{1}{2}\left( 1+\frac{1}{2^2}\right) \,(\hbox {From}\,~\texttt{QRF} \,~\hbox{and using}~\hbox{Equation}\,1)\\&=S_a^{r+1}[i]\oplus S_c^{r+1}[i] \oplus S_d^{r+1}[i-1,i,i+8]\\&\quad \hbox {holds with probability}\,\frac{1}{2}\left( 1+\frac{1}{2^2}\right) . \end{aligned} \end{aligned}$$
4. $$\begin{aligned} \begin{aligned} S_d^r[i-1,i]&=S_a^{r+\frac{1}{2}}[i-1,i]\oplus S_d^{r+\frac{1}{2}}[i+15,i+16]\,~\hbox{holds with probability}\,1\\&\quad (\hbox {From}\,~\texttt{QRF})\\&=S_a^{r+1}[i]\oplus S_b^{r+1}[i+7]\oplus S_c^{r+1}[i]\oplus S_a^{r+1}[i+15,i+16]\\&\quad \oplus S_d^{r+1}[i+23,i+24]\,~\hbox{holds with probability}\,\frac{1}{2}\left( 1+\frac{1}{2}\right) \\&\quad (\hbox {From}\,~\texttt{QRF} \,~\hbox{and using}\,~Lemma\,3)\\&=S_a^{r+1}[i,i+15,i+16]\oplus S_b^{r+1}[i+7]\oplus S_c^{r+1}[i]\oplus S_d^{r+1}[i+23,i+24]\\&\quad \hbox {holds with probability}\,\frac{1}{2}\left( 1+\frac{1}{2}\right) . \end{aligned} \end{aligned}$$

Hence, the proof is completed. Q.E.D.

The above discussed Lemma 4 helps us identify the less number of active bits in the 7th round for the linear approximation from 6th round to 7th round that we use in Result 4. Further, this helps us extend the distinguisher for the 7.25th round of the cipher.

**Figure 4:**
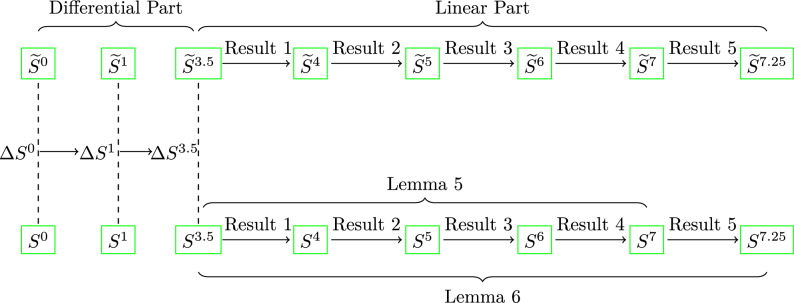
Diagram of overall attack framework.

### Distinguisher for 7 rounds ChaCha permutation

Here we explain the differential-linear distinguisher for the 7 rounds ChaCha permutation using the above-discussed 3.5 rounds differential distinguisher in “Differential distinguisher for the 3.5 rounds ChaCha” section.

In the following, we discuss a few results that will show us the light for the path of linear approximation for 7 rounds of the permutation. The overall attack is framed in Fig. 4.

#### Result 1

The following relation

$$\begin{aligned} S_8^{3.5}[0]=S_8^4[0]\oplus S_{13}^4[0], \end{aligned}$$

holds with probability 1..

#### Proof

Since $\mathrm{Car}(x,y)[0]=0$. The proof directly follows from the QRF for $c=8$, $d=13$ and $r=3.~\text{Q.E.D.}$

The following Result 2 provides the linear approximation from the 4th round to the 5th round.

#### Result 2

The following relation

$$\begin{aligned} S_8^4[0]\oplus S_{13}^4[0]=S_0^5[0]\oplus S_8^5[0] \oplus S_{12}^5[0,8]\oplus S_1^5[0,16]\oplus S_5^5[7] \oplus S_9^5[0] \oplus S_{13}^5[24], \end{aligned}$$

holds with probability 1.

#### Proof

Here the linear relation is between the 4th round and the 5th round among the bits. The round update from 4th round to 5th round, odd round, i.e., column round occurs. So, we can write from the $\texttt{QRF}$ that operates on $(S_0^4, S_4^4, S_8^4, S_{12}^4)$

$$\begin{aligned} S_8^4[0]&=S_8^{4.5}[0]\oplus S_{12}^{4.5}[0]\\&=S_8^{5}[0]\oplus S_{12}^{5}[0] \oplus S_0^5[0] \oplus S_{12}^5[8] \\&=S_0^5[0]\oplus S_8^5[0] \oplus S_{12}^5[0,8]. \end{aligned}$$

In the same way for the column $(S_1^4, S_5^4, S_9^4, S_{13}^4)$, we have

$$\begin{aligned} S_{13}^4[0]&=S_1^{4.5}[0]\oplus S_{13}^{4.5}[16]\\&=S_1^5[0]\oplus S_5^5[7]\oplus S_9^[0]\oplus S_1^5[16]\oplus S_{13}^5[24]\\&=S_1^5[0,16]\oplus S_5^5[7] \oplus S_9^5[0] \oplus S_{13}^5[24]. \end{aligned}$$

Therefore, combining the above two linear relations, we get that the required linear relation

$$\begin{aligned} S_8^4[0]\oplus S_{13}^4[0]=S_0^5[0]\oplus S_8^5[0] \oplus S_{12}^5[0,8]\oplus S_1^5[0,16]\oplus S_5^5[7] \oplus S_9^5[0] \oplus S_{13}^5[24], \end{aligned}$$

holds with probability 1. Q.E.D.

Till now, we have constructed linear approximation up to the 5th round. We derive the linear approximation from the 5th round to the 6th round as follows.

#### Result 3

The following linear relation

3 $$\begin{aligned}{}&S_0^5[0]\oplus S_8^5[0] \oplus S_{12}^5[0,8]\oplus S_1^5[0,16]\oplus S_5^5[7] \oplus S_9^5[0] \oplus S_{13}^5[24]\nonumber \\&\quad =S_0^6[0]\oplus S_8^6[0,23,24]\oplus S_{12}^6[15,16,24] \oplus \nonumber \\ {}&S_1^6[8,24] \oplus S_5^6[7,19,26]\oplus S_9^6[0] \oplus S_{13}^6[0,8,16]\oplus \nonumber \\&S_2^6[0,8,24]\oplus S_6^6[2,3,14,15,19,22,23]\oplus S_{10}^6[7,12,19]\oplus S_{14}^6[0,8]\oplus \nonumber \\&S_3^6[0] \oplus S_7^6[30,31] \oplus S_{11}^6[0,7,8,12,27,28]\oplus S_{15}^6[0,6,7], \end{aligned}$$

holds with probability $\frac{1}{2}(1+\frac{1}{2^6}).$

#### Proof

We have to prove the linear relations between some bits of the state after the 4th round with some bits of the state after the 5th round. Here the bits involved in the linear relation of the words $S_0^5, S_1^5, S_5^5, S_8^5, S_9^5, S_{12}^5, S_{13}^5$ of the state matrix after 5th round. The state update from 5th round to 6th round, even round, i.e., diagonal round occurs. So, the QRF operates on the tuples $(S_0^5,S_5^5,S_{10}^5,S_{15}^5),(S_1^5,S_6^5,S_{11}^5,S_{12}^5),(S_2^5,S_7^5,S_{8}^5,S_{13}^5),(S_3^5,S_4^5,S_{9}^5,S_{14}^5)$ for the round update. Now for the diagonal $(S_0^5,S_5^5,S_{10}^5,S_{15}^5)$, we have from the QRF

$$\begin{aligned} S_0^5[0]&=S_0^{5.5}[0]\oplus S_5^5[0]\\&=S_0^6[0]\oplus S_5^{5.5}[0,12]\oplus S_{10}^{5.5}[0]\\&=S_0^6[0]\oplus S_5^6[7,19]\oplus S_{10}^6[12]\oplus S_{15}^6[0]. \end{aligned}$$

Using Lemma 1 we get that the linear relation

$$\begin{aligned} S_5^5[7]=S_5^6[26]\oplus S_{10}^6[7,19] \oplus S_{15}^6[6,7], \end{aligned}$$

holds with probability $\frac{1}{2}(1+\frac{1}{2}).$

For the diagonal $(S_1^5,S_6^5,S_{11}^5,S_{12}^5)$, we get from the QRF

$$\begin{aligned}{}&S_1^5[0]=S_1^6[0]\oplus S_6^6[7,19] \oplus S_{11}^6[12] \oplus S_{12}^6[0],\\&S_{12}^5[0]=S_1^6[0,16]\oplus S_6^6[7]\oplus S_{11}^6[0] \oplus S_{12}^6[24]. \end{aligned}$$

Using Lemma 1 we have that the linear relations

$$\begin{aligned} S_1^5[16]=S_1^6[16]\oplus S_6^6[2,3,22,23]\oplus S_{11}^6[27,28]\oplus S_{12}^6[15,16], \end{aligned}$$

and

$$\begin{aligned} S_{12}^5[8]=S_1^6[8,24]\oplus S_6^6[14,15] \oplus S_{11}^6[7,8]\oplus S_{12}^6[0], \end{aligned}$$

hold with probabilites $\frac{1}{2}(1+\frac{1}{2^3})$ and $\frac{1}{2}(1+\frac{1}{2})$ respectively.

For the diagonal $(S_2^5,S_7^5,S_{8}^5,S_{13}^5)$, we get from the QRF

$$\begin{aligned} S_8^5[0]=S_2^6[0]\oplus S_8^6[0] \oplus S_{13}^6[0,8]. \end{aligned}$$

Also, using Lemma 1 we get that the linear relation

$$\begin{aligned} S_{13}^5[24]=S_2^6[8,24]\oplus S_7^6[30,31] \oplus S_8^6[23,24] \oplus S_{13}^6[16], \end{aligned}$$

holds with probability $\frac{1}{2}(1+\frac{1}{2}).$

For the diagonal $(S_3^5,S_4^5,S_{9}^5,S_{14}^5)$, we get from the QRF

$$\begin{aligned} S_9^5[0]=S_3^6[0]\oplus S_9^6[0] \oplus S_{14}^6[0,8]. \end{aligned}$$

Therefore, combing the above obtained linear relations and using Pilling-up Lemma^29^, we get the required result. Q.E.D.

The following Result 4 presents the linear approximation from the 6th round to the 7th round.

#### Result 4

The following linear relation

4 $$\begin{aligned}{}&S_0^6[0]\oplus S_8^6[0,23,24]\oplus S_{12}^6[15,16,24]\oplus \nonumber \\&S_1^6[8,24] \oplus S_5^6[7,19,26]\oplus S_9^6[0]\oplus S_{13}^6[0,8,16]\oplus \nonumber \\&S_2^6[0,8,24]\oplus S_6^6[2,3,14,15,19,22,23]\oplus S_{10}^6[7,12,19] \oplus S_{14}^6[0,8]\oplus \nonumber \\&S_3^6[0] \oplus S_7^6[30,31] \oplus S_{11}^6[0,7,8,12,27,28]\oplus S_{15}^6[0,6,7]\oplus \nonumber \\&\quad =S_0^7[0,8,16,31]\oplus S_4^7[7,19,23,30,31] \oplus S_8^7[0,12,16,23]\oplus S_{12}^7[0,7,16,23,24]\oplus \nonumber \\&S_1^7[0]\oplus S_5^7[6,7,10,11,13,22,23,27,30,31]\oplus S_9^7[3,4,6,8,15,16,19,20,26,31]\oplus \nonumber \\&S_{13}^7[6,8,18,19,23,25,26]\oplus S_2^7[6,7,11,12,16,18,19]\oplus \nonumber \\&S_6^7[1,2,6,9,11,19,21,22,26,27,30,31]\oplus S_{10}^7[0,2,3,4,8,14,19,20,23,26,27,31] \oplus \nonumber \\&S_{14}^7[3,6,8,11,12,14,19,20,26,27]\oplus S_3^7[0,7,8,11,12,16,22,23,28]\oplus \nonumber \\&S_7^7[14,17,18,19]\oplus S_{11}^7[7,8,10,11,28,31] \oplus S_{15}^7[0,4,7,11,12,16,19,20,24,27,28,30], \end{aligned}$$

holds with probability $\frac{1}{2}(1+\frac{1}{2^{40}}).$

#### Proof

Here the linear approximation is from round 6 to round 7. So, the column round operates on the state matrix, i.e., the quarter round function applies to each column of the state matrix to update the round. After the 6 round, we divide the bits of the state matrix column-wise into four groups as follows.

(i) Column I: $$S_0^6[0]$$, $$S_8^6[0,23,24]$$ and $$S_{12}^6[15,16,24]$$.
(ii) Column II: $S_1^6[8,24]$, $S_5^6[7,19,26]$, $S_9^6[0]$ and $S_{13}^6[0,8,16]$.
(iii) Column III: $S_2^6[0,8,24]$, $S_6^6[2,3,14,15,19,22,23]$, $S_{10}^6[7,12,19]$ and $S_{14}^6[0,8]$.
(iv) Column IV: $S_3^6[0]$, $S_7^6[30,31]$, $S_{11}^6[0,7,8,12,27,28]$ and $S_{15}^6[0,6,7]$.

Now we discuss how to find the linear approximations for each group of bits in the following.

(i) For the group of bits $\underline{Column\,I}$, the involved column is $(S_0^6,S_4^6,S_8^6,S_{12}^6).$ We get the following linear approximations from QRF and using the above-discussed Lemmas.
    $$\begin{aligned} \begin{aligned} S_0^6[0]=S_0^7[0]\oplus S_4^7[7,19] \oplus S_8^7[12] \oplus S_{12}^7[0]\,\hbox {holds with probability} \,1.\end{aligned} \end{aligned}$$
    $$\begin{aligned} \begin{aligned} S_8^6[0]=S_0^7[0]\oplus S_8^7[0]\oplus S_{12}^7[0,8]\,\hbox {holds with probability}\,1. \end{aligned} \end{aligned}$$
    $$\begin{aligned} \begin{aligned} S_8^6[23,24]=S_0^7[24]\oplus S_8^7[24] \oplus S_{12}^7[0,23,24]\,\hbox {holds with probability}\,\frac{1}{2}\left( 1+\frac{1}{2^2}\right) .\end{aligned} \end{aligned}$$
    $$\begin{aligned} \begin{aligned} S_{12}^6[15,16]&=S_0^7[0,16,31]\oplus S_4^7[23] \oplus S_8^7[16]\oplus S_{12}^7[7,8]\\&\quad \hbox {holds with probability}\,\frac{1}{2}\left( 1+\frac{1}{2}\right) . \end{aligned} \end{aligned}$$
    $$\begin{aligned} \begin{aligned} S_{12}^6[24]&=S_0^7[8,24]\oplus S_4^7[30,31] \oplus S_8^7[23,24]\oplus S_{12}^7[16]\\&\quad \hbox {holds with probability}\,\frac{1}{2}\left( 1+\frac{1}{2}\right) . \end{aligned} \end{aligned}$$
  Combining the above obtained linear approximations of Column I and using Pilling-up Lemma^29^, we get the following linear approximation
  5 $$\begin{aligned} S_0^6[0]\oplus S_8^6[0,23,24]\oplus S_{12}^6[15,16,24]&=S_0^7[0,8,16,31]\oplus S_4^7[7,19,23,30,31]\nonumber \\&\oplus S_8^7[0,12,16,23] \oplus S_{12}^7[0,7,16,23,24], \end{aligned}$$
  that holds with probability $\frac{1}{2}(1+\frac{1}{2^{4}}).$
(ii) For the group of bits $\underline{Column\,II}$, the involved column is $(S_1^6,S_5^6,S_9^6,S_{13}^6).$ We have the linear approximations as follows.
    $$\begin{aligned} \begin{aligned} S_1^6[8]&=S_1^7[8]\oplus S_5^7[14,15,26,27]\oplus S_9^7[19,20] \oplus S_{13}^7[7,8]\\&\quad \hbox {holds with probability}\,\frac{1}{2}\left( 1+\frac{1}{2^3}\right) .\end{aligned} \end{aligned}$$
    $$\begin{aligned} \begin{aligned} S_1^6[24]&=S_1^7[24]\oplus S_5^7[10,11,30,31] \oplus S_9^7[3,4] \oplus S_{13}^7[23,24]\\&\quad \hbox {holds with probability}\,\frac{1}{2}\left( 1+\frac{1}{2^3}\right) . \end{aligned} \end{aligned}$$
    $$\begin{aligned} \begin{aligned} S_5^6[7]=S_5^7[26]\oplus S_9^7[7,19]\oplus S_{13}^7[6,7]\,\hbox {holds with probability}\,\frac{1}{2}\left( 1+\frac{1}{2}\right) . \end{aligned} \end{aligned}$$
    $$\begin{aligned} \begin{aligned} S_5^6[19]=S_5^7[6]\oplus S_9^7[19,31]\oplus S_{13}^7[18,19]\,\hbox {holds with probability}\,\frac{1}{2}\left( 1+\frac{1}{2}\right) .\end{aligned} \end{aligned}$$
    $$\begin{aligned} \begin{aligned} S_5^6[26]=S_5^7[13]\oplus S_9^7[6,26]\oplus S_{13}^7[25,26]\,\hbox {holds with probability}\,\frac{1}{2}\left( 1+\frac{1}{2}\right) .\end{aligned} \end{aligned}$$
    $$\begin{aligned} \begin{aligned} S_9^6[0]=S_1^7[0]\oplus S_9^7[0] \oplus S_{13}^7[0,8]\,\hbox {holds with probability}\,1. \end{aligned} \end{aligned}$$
    $$\begin{aligned} \begin{aligned} S_{13}^6[0]=S_1^7[0,16]\oplus S_5^7[7] \oplus S_9^7[0]\oplus S_{13}^7[24]\,\hbox {holds with probability}\,1.\end{aligned} \end{aligned}$$
    $$\begin{aligned} \begin{aligned} S_{13}^6[8]&=S_1^7[8,24]\oplus S_5^7[14,15] \oplus S_9^7[7,8] \oplus S_{13}^7[0]\\&\quad \hbox {holds with probability}\,\frac{1}{2}\left( 1+\frac{1}{2}\right) .\end{aligned} \end{aligned}$$
    $$\begin{aligned} \begin{aligned} S_{13}^6[16]&=S_1^7[0,16]\oplus S_5^7[22,23] \oplus S_9^7[15,16] \oplus S_{13}^7[8]\\&\quad \hbox {holds with probability}\,\frac{1}{2}\left( 1+\frac{1}{2}\right) .\end{aligned} \end{aligned}$$
  Now a combination of the above linear approximations of Column II and using the Pilling-up Lemma^29^, we get the following linear approximation
  6 $$\begin{aligned}{}&S_1^6[8,24] \oplus S_5^6[7,19,26]\oplus S_9^6[0] \oplus S_{13}^6[0,8,16]\nonumber \\&\quad =S_1^7[0]\oplus S_5^7[6,7,10,11,13,22,23,27,30,31] \oplus \nonumber \\&S_9^7[3,4,6,8,15,16,19,20,26,31]\oplus S_{13}^7[6,7,18,19,23,25,26], \end{aligned}$$
  that holds with probability $\frac{1}{2}(1+\frac{1}{2^{11}}).$
(iii) For the bits involved in the group $\underline{Column\,III}$, the corresponding column is $(S_2^6,S_6^6,S_{10}^6,S_{14}^6).$ We get the following linear approximations.
    $$\begin{aligned} \begin{aligned} S_2^6[0]=S_2^7[0]\oplus S_6^7[7,19] \oplus S_{10}^7[12] \oplus S_{14}^7[0]\,\hbox {holds with probability}\,1. \end{aligned} \end{aligned}$$
    $$\begin{aligned} \begin{aligned} S_2^6[8]&=S_2^7[8]\oplus S_6^7[14,15,26,27]\oplus S_{10}^7[19,20]\oplus S_{14}^7[7,8]\\&\quad \hbox {holds with probability}\,\frac{1}{2}\left( 1+\frac{1}{2^3}\right) . \end{aligned} \end{aligned}$$
    $$\begin{aligned} \begin{aligned} S_2^6[24]&=S_2^7[24]\oplus S_6^7[10,11,30,31]\oplus S_{10}^7[3,4]\oplus S_{14}^7[23,24]\\&\quad \hbox {holds with probability}\,\frac{1}{2}\left( 1+\frac{1}{2^3}\right) . \end{aligned} \end{aligned}$$
    $$\begin{aligned} \begin{aligned} S_6^6[2,3]=S_6^7[21,22]\oplus S_{10}^7[3,14,15]\oplus S_{14}^7[3]\,\hbox {holds with probability}\,\frac{1}{2}\left( 1+\frac{1}{2}\right) . \end{aligned} \end{aligned}$$
    $$\begin{aligned} \begin{aligned} S_6^6[14,15]&=S_6^7[1,2]\oplus S_{10}^7[15,26,27]\oplus S_{14}^7[15]\\&\quad \hbox {holds with probability}\,\frac{1}{2}\left( 1+\frac{1}{2}\right) .\end{aligned} \end{aligned}$$
    $$\begin{aligned} \begin{aligned} S_6^6[22,23]&=S_6^7[9,10]\oplus S_{10}^7[2,3,23]\oplus S_{14}^7[23]\,\hbox {holds with probability}\,\frac{1}{2}\left( 1+\frac{1}{2}\right) .\end{aligned} \end{aligned}$$
    $$\begin{aligned} \begin{aligned} S_6^6[19]=S_6^7[6]\oplus S_{10}^7[19,31]\oplus S_{14}^7[18,19]\,\hbox {holds with probability}\,\frac{1}{2}\left( 1+\frac{1}{2}\right) .\end{aligned} \end{aligned}$$
    $$\begin{aligned} \begin{aligned} S_{10}^6[7]=S_2^7[6,7]\oplus S_{10}^7[7]\oplus S_{14}^7[6,7,14,15]\,\hbox {holds with probability}\,\frac{1}{2}\left( 1+\frac{1}{2^2}\right) .\end{aligned} \end{aligned}$$
    $$\begin{aligned} \begin{aligned} S_{10}^6[12]&=S_2^7[11,12]\oplus S_{10}^7[12]\oplus S_{14}^7[11,12,19,20]\\&\quad \hbox {holds with probability}\,\frac{1}{2}\left( 1+\frac{1}{2^2}\right) .\end{aligned} \end{aligned}$$
    $$\begin{aligned} \begin{aligned} S_{10}^6[19]&=S_2^7[18,19]\oplus S_{10}^7[19]\oplus S_{14}^7[18,19,26,27]\\&\quad \hbox {holds with probability}\,\frac{1}{2}\left( 1+\frac{1}{2^2}\right) . \end{aligned} \end{aligned}$$
    $$\begin{aligned} \begin{aligned} S_{14}^6[0]=S_2^7[0,16]\oplus S_6^7[7] \oplus S_{10}^7[0]\oplus S_{14}^7[24]\,\hbox {holds with probability}\,1.\end{aligned} \end{aligned}$$
    $$\begin{aligned} \begin{aligned} S_{14}^6[8]&=S_2^7[8,24]\oplus S_6^7[14,15] \oplus S_{10}^7[7,8]\oplus S_{14}^7[0]\\&\quad \hbox {holds with probability}\,\frac{1}{2}\left( 1+\frac{1}{2}\right) .\end{aligned} \end{aligned}$$
  Now, by combining the above linear approximations of Column III and using the Pilling-up Lemma^29^, we get the following linear approximation
  7 $$\begin{aligned}{}&S_2^6[0,8,24]\oplus S_6^6[2,3,14,15,19,22,23]\oplus S_{10}^6[7,12,19]\oplus S_{14}^6[0,8]\nonumber \\&\quad =S_2^7[6,7,11,12,16,18,19]\oplus S_6^7[1,2,6,9,11,19,21,22,26,27,30,31]\oplus \nonumber \\&S_{10}^7[0,2,3,4,8,14,19,20,23,26,27,31]\oplus S_{14}^7[3,6,8,11,12,14,19,20,26,27], \end{aligned}$$
  that holds with probability $\frac{1}{2}(1+\frac{1}{2^{17}}).$
(iv) For the bits presented in the group $\underline{Column\,IV}$, the corresponding column is $(S_3^6,S_7^6,S_{11}^6,S_{15}^6).$ We have the linear approximations as follows.
    $$\begin{aligned} \begin{aligned} S_3^6[0]=S_3^7[0]\oplus S_7^7[7,19]\oplus S_{11}^7[12]\oplus S_{15}^7[0]\,\hbox {holds with probability}\,1.\end{aligned} \end{aligned}$$
    $$\begin{aligned} \begin{aligned} S_7^6[30,31]&=S_7^7[17,18] \oplus S_{11}^7[10,11,31]\oplus S_{15}^7[31]\\&\quad \hbox {holds with probability}\,\frac{1}{2}\left( 1+\frac{1}{2}\right) .\end{aligned} \end{aligned}$$
    $$\begin{aligned} \begin{aligned} S_{11}^6[0]=S_3^7[0]\oplus S_{11}^7[0]\oplus S_{15}^7[0,8]\,\hbox {holds with probability}\,1.\end{aligned} \end{aligned}$$
    $$\begin{aligned} \begin{aligned} S_{11}^6[12]&=S_3^7[11,12]\oplus S_{11}^7[12]\oplus S_{15}^7[11,12,19,20]\\&\quad \hbox {holds with probability}\,\frac{1}{2}\left( 1+\frac{1}{2^2}\right) .\end{aligned} \end{aligned}$$
    $$\begin{aligned} \begin{aligned} S_{11}^6[7,8]&=S_3^7[8] \oplus S_{11}^7[8]\oplus S_{15}^7[7,8,16]\,\hbox {holds with probability}\,\frac{1}{2}\left( 1+\frac{1}{2^2}\right) .\end{aligned} \end{aligned}$$
    $$\begin{aligned} \begin{aligned} S_{11}^6[27,28]&=S_3^7[28] \oplus S_{11}^7[28]\oplus S_{15}^7[4,27,28]\\&\quad \hbox {holds with probability}\,\frac{1}{2}\left( 1+\frac{1}{2^2}\right) .\end{aligned} \end{aligned}$$
    $$\begin{aligned} \begin{aligned} S_{15}^6[0]=S_3^7[0,16]\oplus S_7^7[7]\oplus S_{11}^7[0] \oplus S_{15}^7[24]\,\hbox {holds with probability}\,1.\end{aligned} \end{aligned}$$
    $$\begin{aligned} \begin{aligned} S_{15}^6[6,7]&=S_3^7[7,22,23]\oplus S_7^7[14]\oplus S_{11}^7[7]\oplus S_{15}^7[30,31]\\&\quad \hbox {holds with probability}\,\frac{1}{2}\left( 1+\frac{1}{2}\right) . \end{aligned} \end{aligned}$$
  Now by combining the above linear approximations of Column IV and using the Pilling-up Lemma^29^, we get the following linear approximation
  8 $$\begin{aligned}{}&S_3^6[0] \oplus S_7^6[30,31] \oplus S_{11}^6[0,7,8,12,27,28]\oplus S_{15}^6[0,6,7]\nonumber \\&\quad =S_3^7[0,7,8,11,12,16,22,23,28]\oplus S_7^7[14,17,18,19]\oplus \nonumber \\&S_{11}^7[7,8,10,11,28,31] \oplus S_{15}^7[4,7,11,12,16,19,20,24,27,28,30], \end{aligned}$$
  that holds with probability $\frac{1}{2}(1+\frac{1}{2^{8}}).$

Finally, by combing the above four groups of linear approximations Result 5, Result 6, Result 7 and Result 8, we get the required result. Q.E.D.

By combing the above-discussed results Result 1, Result 2, Result 3 and Result 4, we get the following Lemma 5 that gives the linear approximation between an active bit of the 3.5th round and multiple active bits of the 7th round of the permutation.

#### Lemma 5

The following path of linear approximation from 3.5 rounds to 7 rounds of ChaCha

9 $$\begin{aligned} S_8^{3.5}[0]&=S_0^7[0,8,16,31]\oplus S_4^7[7,19,23,30,31] \oplus S_8^7[0,12,16,23]\oplus S_{12}^7[0,7,16,23,24]\oplus \nonumber \\&S_1^7[0]\oplus S_5^7[6,7,10,11,13,22,23,27,30,31]\oplus S_9^7[3,4,6,8,15,16,19,20,26,31]\oplus \nonumber \\&S_{13}^7[6,8,18,19,23,25,26]\oplus S_2^7[6,7,11,12,16,18,19]\oplus \nonumber \\&S_6^7[1,2,6,9,11,19,21,22,26,27,30,31]\oplus S_{10}^7[0,2,3,4,8,14,19,20,23,26,27,31] \oplus \nonumber \\&S_{14}^7[3,6,8,11,12,14,19,20,26,27]\oplus S_3^7[0,7,8,11,12,16,22,23,28]\oplus \nonumber \\&S_7^7[14,17,18,19]\oplus S_{11}^7[7,8,10,11,28,31] \oplus S_{15}^7[4,7,11,12,16,19,20,24,27,28,30], \end{aligned}$$

holds with probability $\frac{1}{2}(1+\frac{1}{2^{46}}).$

#### Proof

The proof follows from the above-discussed results Result 1, Result 2, Result 3 and Result 4 using Pilling-up Lemma^29^. Q.E.D.

In the following, we present the computational results of the above theoretically obtained linear approximations. There is a gap between some of the theoretical results and computational results because of the independence assumption in the theoretical results.

#### Experiment 1

The linear approximations of Result 1 and Result 2 hold computationally with linear correlations $\epsilon _{l1}=1$ and $\epsilon _{l2}=1$ respectively, which are the same as theoretically obtained linear correlations.

The following Experiment 2 provides the computational result for the Result 3.

#### Experiment 2

The linear approximation of the Result 3 holds computationally with linear correlation $\epsilon _{l3}=0.0209\approx 2^{-5.58}.$ This correlation is confirmed using $2^{40}$ random samples.

We have divided the linear approximation of Result 4 into four groups in the proof. The four groups have four different linear approximations which are Result 5, Result 6, Result 7 and Result 8. We have proved these linear approximations Result 5, Result 6, Result 7 and Result 8 theoretically in the Result 4 and the corresponding computational results Experiment 3, Experiment 4 are as follows.

#### Experiment 3

The linear approximations of the equations Result 5 and Result 6 hold computationally with linear correlations $\epsilon _{l4}=0.083\approx 2^{-3.59}$ and $\epsilon _{l5}=0.0027\approx 2^{-8.53}$ respectively. These correlations are confirmed using $2^{40}$ random samples.

#### Experiment 4

The linear relation of the equations Result 7 and Result 8 hold computationally with linear correlations $\epsilon _{l6}=0.00029\approx 2^{-11.75}$ and $\epsilon _{l7}=0.0092\approx 2^{-6.76}$ respectively. These correlations are confirmed using $2^{40}$ random samples.

#### Remark 1

In Experiment 3 and Experiment 4 one can see that there is a significant difference between theoretically and computationally obtained correlations. Theoretical proofs assume the hypothesis of independence through the Piling-Up Lemma, leading to some discrepancies when compared to experimental results. The large number of samples used in the experiments resulted in more accurate results than the theoretical ones. Therefore, computational results are used when determining attack complexities. Further research may be conducted to understand why ChaCha exhibits this behavior.

Now we discuss the complexity analysis of the improved 7 rounds distinguisher.

#### Complexity

We experimented with all the required linear approximations to compute the differential-linear distinguisher for 7 rounds ChaCha permutation. Then using the above experiments Experiment 1, Experiment 2, Experiment 3 and Experiment 4, we get the linear correlation $\epsilon _l=\epsilon _{l1}\epsilon _{l2}\epsilon _{l3}\epsilon _{l4}\epsilon _{l5}\epsilon _{l6}\epsilon _{l7}\approx 2^{-36.21}$ and also we know the differential correlation $\epsilon _d\approx 2^{-28.65}.$ Then we calculate $\epsilon _d\epsilon _l^2\approx 2^{-101}$ as the differential-linear correlation for the 7 rounds of the permutation. Hence the data and time complexity for 7 rounds differential-linear distinguisher is $2^{207}$ as the attack has to repeat $2^5$ times on average.

##### Remark 2

In distinguishing attacks, the adversary generates ciphertexts from chosen plaintexts using the encryption machinery under the fixed secret key. In the case of the ChaCha permutation, the adversary chose the IVs to generate outputs and then tried to distinguish the permutation from a random permutation. But the total size of IV in the initial state of ChaCha is 128 bits. Nevertheless, in the above complexity analysis, we have seen that the required data is $2^{207}.$ In the attack, one may not fix the secret key; otherwise, it is not possible to generate the huge data for the attack. So, we fix only the 64 bits of the secret key corresponding to the input difference column $(S^0_2, S^0_6, S^0_{10}, S^0_{14}).$ In the papers^24,26^, the authors have not clearly mentioned how the data is generated to mount the attack. In those papers^24,26^, the data complexities were $2^{224}$ and $2^{214}$ respectively. If the whole secret key is fixed for generating the data, this is impossible because IV size is 128 bits. We have pointed out this issue.

##### Note 1

Here, we explain that the previous multi-bits distinguishing attacks on the reduced round of the cipher ChaCha are invalid. These attacks are valid for ChaCha permutation only. The output of *R*-round ChaCha permutation is $P=S^R$, and the output or key stream of *R*-round ChaCha cipher is $Z=S^0\boxplus S^R$. In the attack procedure, the linear relation is obtained with the permutation output, not with the key stream of the cipher. The multiple bits involved in the permutation output differ from the cipher output/key stream. Suppose $S'^0$ be another initial state, where $S'^0=S^0\oplus \Delta S^0$ and $S'^R$ is the corresponding updated state after *R*-round, i.e., $P'=S'^R$ the permutation output after *R*-round. The key stream is $Z'=S'^0\oplus S'^R.$ The targeted multiple bits involved in the permutation output difference $\Delta P=P\oplus P'=S^R\oplus S'^R$ are not identical with the bits involved in the key stream difference $\Delta Z=Z\oplus Z'= (S^0\boxplus S^R)\oplus (S'^0\boxplus S'^R)$ for $R=7$.

##### Remark 3

We use a heuristic approach to find linear approximations. Here we have presented the linear approximation (Lemma 5) starting from the bit (8, 0) of the 3.5th round to multiple bits in the 7th round. The linear approximation holds with the same probability if we start from the position (9, 0) or (10, 0) or (11, 0) of the 3.5th round instead of the position (8, 0) to different multiple bits of the 7th rounds. The diagonal round is operated for the update from 3rd to 4th round. If we consider a diagonal of the state matrix of the cipher according to diagonal round with indices (*a*, *b*, *c*, *d*), then from the QRF, we know that the word with index *a* is updated first, the word with index *d* is updated second, then the word with index *c* is updated, and in the last, the word with index *b* is updated. In our attack framework, we start the linear approximation from the 0th bit of the word with index *c* after the 3.5 rounds. In the existing works of differential-linear distinguishers, the authors started with the 0th bit of the word with index *a* after the 3.5 rounds.

##### Remark 4

One natural question arises in mind: why have we not started with the other bit positions instead of the 0th bit? If we use any bit positions other than 0th bit, then the linear approximation from the 3.5 rounds to the 4 rounds involves 3 bits; for example, suppose $S^{3.5}_c[i]=S^{4}_c[i]\oplus S^4_d[i]\oplus S^4_d[i-1]$ for $i>0$ and the linear approximation holds with probability 0.75, but for the 0th bit, the number of involved bits is two, and it holds with probability 1. Another question is why we have not used the words with indices *d* and *b*. If we use the 0th bit of the word with index *d* after the 3.5 rounds, we get the linear approximation $S^{3.5}_d[0]=S^4_a[0]\oplus S^4_d[8]$ that holds with probability 1. But for the bit $S^4_d[8]$, the further linear approximations up to 7th hold with very less probability that does not improve the latest work. Also, after the 3.5 rounds, we cannot find observable bias for the 0th bit of the word with index *b*; this is why we do not consider the 0th bit of the word with index *b*.

### Distinguisher for 7.25 rounds ChaCha permutation

Here we discuss the extension of differential-linear distinguisher from the 7th round to the 7.25th round of ChaCha permutation.

The quarter-round function $(\texttt{QRF} _{0.25})$ for 0.25 round is given below:

$$\begin{aligned}{}&S_a^{r+\frac{1}{4}} =S_a^{r}\boxplus S_b^{r},\\&S_d^{r+\frac{1}{4}}=(S_d^{r}\oplus S_a^{r+\frac{1}{4}})\lll 16,\\&S_c^{r+\frac{1}{4}} = S_c^{r}, \\ {}&S_b^{r+\frac{1}{4}}=S_b^{r}, \end{aligned}$$

i.e.,

$$\begin{aligned} \texttt{QRF} _{0.25}(S_a^{r},S_b^{r},S_c^{r},S_d^{r})\rightarrow (S_a^{r+1\frac{1}{4}},S_b^{r+\frac{1}{4}},S_c^{r+\frac{1}{4}},S_d^{r+\frac{1}{4}}). \end{aligned}$$

Therefore, in the 0.25th round update of the state, for each 4-tuple $(S_a^{r},S_b^{r},S_c^{r},S_d^{r})$ only the words $S_a^r$ and $S_d^r$ is updated and other two words $S_b^r$ and $S_c^r$ remain unchanged as describe above. So, in the 0.25 round update, 8 words among 16 words of the state matrix are updated.

Now from the $\texttt{QRF} _{0.25}$, we get the following relations using the approximation of Eq. (1).

- 10 $$\begin{aligned} \begin{aligned} S_a^r[i]=S_a^{r+\frac{1}{4}}[i]\oplus S_b^{r+\frac{1}{4}}[i-1,i] \,\hbox {holds with probability}\,\frac{1}{2}\left( 1+\frac{1}{2}\right) \,\hbox {for}\,i>0.\end{aligned} \end{aligned}$$
- 11 $$\begin{aligned} \begin{aligned} S_a^r[i-1,i]=S_a^{r+\frac{1}{4}}[i]\oplus S_b^{r+\frac{1}{4}}[i] \,\hbox {holds with probability}\,\frac{1}{2}\left( 1+\frac{1}{2}\right) \,\hbox {for}\,i>0.\end{aligned} \end{aligned}$$
- 12 $$\begin{aligned} \begin{aligned} S_d^r[i]=S_a^{r+\frac{1}{4}}[i]\oplus S_d^r[i+16]\,\hbox {holds with probability}\,1 \,\hbox {for}\,i\ge 0.\end{aligned} \end{aligned}$$

The round update from 7th round to 7.25th round ChaCha diagonal round occurs. The following linear approximation forms the probabilistic linear relation between the 7th round and the 7.25th round.

#### Result 5

The following linear approximation

13 $$\begin{aligned}{}&S_0^7[0,8,16,31]\oplus S_5^7[6,7,10,11,13,22,23,27,30,31]\oplus \nonumber \\&S_{10}^7[0,2,3,4,8,14,19,20,23,26,27,31] \oplus S_{15}^7[4,7,11,12,16,19,20,24,27,28,30] \oplus \nonumber \\&S_1^7[0]\oplus S_6^7[1,2,6,9,11,19,21,22,26,27,30,31]\oplus S_{11}^7[7,8,10,11,28,31] \oplus \nonumber \\&S_{12}^7[0,7,16,23,24]\oplus S_2^7[6,7,11,12,16,18,19]\oplus S_7^7[14,17,18,19]\oplus \nonumber \\&S_8^7[0,12,16,23]\oplus S_{13}^7[6,8,18,19,23,25,26]\oplus S_3^7[0,7,8,11,12,16,22,23,28]\oplus \nonumber \\&S_4^7[7,19,23,30,31]\oplus S_9^7[3,4,6,8,15,16,19,20,26,31]\oplus S_{14}^7[3,6,8,11,12,14,19,20,26,27]\nonumber \\&\quad = S_0^{7.25}[0,4,7,8,11,12,19,20,24,27,28,30,31]\oplus S_5^{7.25}[0,6,8,10,11,13,15,16,22,23,27]\oplus \nonumber \\&S_{10}^{7.25}[0,2,3,4,8,14,19,20,23,26,27,31] \oplus S_{15}^{7.5}[0,3,4,8,11,12,14,16,20,23,27,28] \oplus \nonumber \\&S_1^{7.25}[7,16,23,24]\oplus S_6^{7.25}[0,1,2,6,9,11,19,21,22,26,27,30,31] \oplus \nonumber \\&S_{11}^{7.25}[7,8,10,11,28,31] \oplus S_{12}^{7.25}[0,7,8,16,23]\oplus S_2^{7.25}[6,7,8,12,16,18,23,25,26] \oplus \nonumber \\&S_7^{7.25}[7,12,14,15,16,17,18]\oplus S_8^{7.25}[0,12,16,23]\oplus S_{13}^{7.25}[2,3,7,9,10,22,24]\oplus \nonumber \\&S_3^{7.25}[0,3,6,11,14,16,19,20,23,26,27,28]\oplus S_4^{7.25}[0,7,8,12,15,16,19,27,28,30,31]\oplus \nonumber \\&S_9^{7.25}[3,4,6,8,15,16,19,20,26,31]\oplus S_{14}^{7.25}[3,4,10,11,19,22,24,27,28,30], \end{aligned}$$

holds with probability $\frac{1}{2}(1+\frac{1}{2^{12}}).$

#### Proof

We prove the given linear approximation by dividing the bits of the 7th round into the four groups given below. Since diagonal round occurs for updating the state after the 7th round. So, each group consists of the bits corresponding to each diagonal of the state matrix. The groups are as follows:

(i) Diagonal I: $$S_0^7[0,8,16,31]$$, $$S_5^7[6,7,10,11,13,22,23,27,30,31]$$, $$S_{10}^7[0,2,3,4,8,14,19,20,23,26,27,31]$$ and $$S_{15}^7[4,7,11,12,16,19,20,24,27,28,30].$$
(ii) Diagonal II: $S_1^7[0]$, $S_6^7[1,2,6,9,11,19,21,22,26,27,30,31]$, $S_{11}^7[7,8,10,11,28,31]$ and $S_{12}^7[0,7,16,23,24]$.
(iii) Diagonal III: $S_2^7[6,7,11,12,16,18,19]$, $S_7^7[14,17,18,19]$, $S_8^7[0,12,16,23]$ and $S_{13}^7[6,8,18,19,23,25,26]$.
(iv) Diagonal IV: $S_3^7[0,7,8,11,12,16,22,23,28]$, $S_4^7[7,19,23,30,31]$, $S_9^7[3,4,6,8,15,16,19,20,26,31]$ and $S_{14}^7[3,6,8,11,12,14,19,20,26,27]$.

Now we find the linear approximation for each of the groups one by one to prove the final result.

(i) For the bits of group $\underline{Diagonal\,I}$, we get the linear approximations using Eqs. (10),  (11) and  (12) as follows:
    $$\begin{aligned} \begin{aligned} S_0^7[0,8,16,31]&=S_0^{7.25}[0,8,16,31]\oplus S_5^{7.25}[0,7,8,15,16,30,31]\\&\quad \hbox {holds with probability}\,\frac{1}{2}\left( 1+\frac{1}{2^3}\right) .\end{aligned} \end{aligned}$$
    $$\begin{aligned} \begin{aligned}{}&S_{15}^7[4,7,11,12,16,19,20,24,27,28,30]\\&=S_{0}^{7.25}[4,7,11,12,16,19,20,24,27,28,30] \oplus \\&S_{15}^{7.25}[0,3,4,8,11,12,14,20,23,27,28] \,\hbox {holds with probability}\,1.\end{aligned} \end{aligned}$$
    $$\begin{aligned} \begin{aligned} S_5^7[6,7,10,11,13,22,23,27,30,31]&=S_5^{7.25}[6,7,10,11,13,22,23,27,30,31]\\&\quad \hbox {holds with probability}\,1.\end{aligned} \end{aligned}$$
    $$\begin{aligned} \begin{aligned}{}&S_{10}^7[0,2,3,4,8,14,19,20,23,26,27,31]\\&=S_{10}^{7.25}[0,2,3,4,8,14,19,20,23,26,27,31]\,\hbox {holds with probability}\,1. \end{aligned} \end{aligned}$$
  By combining these above approximations, we get the following linear approximation
  14 $$\begin{aligned}{}&S_0^7[0,8,16,31]\oplus S_5^7[6,7,10,11,13,22,23,27,30,31]\oplus \nonumber \\&S_{10}^7[0,2,3,4,8,14,19,20,23,26,27,31] \oplus \nonumber \\&S_{15}^7[0,4,7,11,12,16,19,20,24,27,28,30]\nonumber \\&\quad = S_0^{7.25}[0,4,7,8,11,12,19,20,24,27,28,30,31]\oplus \nonumber \\&S_5^{7.25}[0,6,8,10,11,13,15,16,22,23,27]\oplus \nonumber \\&S_{10}^{7.25}[0,2,3,4,8,14,19,20,23,26,27,31] \oplus S_{15}^{7.5}[0,3,4,8,11,12,14,16,20,23,27,28]\nonumber \\&\,\hbox {that holds with probability}\,\frac{1}{2}\left( 1+\frac{1}{2^3}\right) . \end{aligned}$$
(ii) For the bits of group $\underline{Diagonal\,II}$, we get the linear approximations using Eqs. (10),  (11) and  (12) as follows:
    $$\begin{aligned} \begin{aligned}S_1^7[0]=S_1^{7.25}[0]\oplus S_6^{7.25}[0]\,\hbox {holds with probability}\,1.\end{aligned} \end{aligned}$$
    $$\begin{aligned} \begin{aligned}S_{12}^7[0,7,16,23,24]&=S_1^{7.25}[0,7,16,23,24]\oplus S_{12}^{7.25}[0,7,8,16,23]\\&\quad \hbox {holds with probability}\,1.\end{aligned} \end{aligned}$$
    $$\begin{aligned} \begin{aligned}{}&S_6^7[1,2,6,9,11,19,21,22,26,27,30,31]\\&\quad =S_6^{7.25}[1,2,6,9,11,19,21,22,26,27,30,31]\,\hbox {holds with probability}\,1.\end{aligned} \end{aligned}$$
    $$\begin{aligned} \begin{aligned}S_{11}^7[7,8,10,11,28,31]&=S_{11}^{7.25}[7,8,10,11,28,31]\\&\quad \hbox {holds with probability}\,1.\end{aligned} \end{aligned}$$
  By combining these above approximations, we get the following linear approximation
  15 $$\begin{aligned}{}&S_1^7[0]\oplus S_6^7[1,2,6,9,11,19,21,22,26,27,30,31]\oplus \nonumber \\&S_{11}^7[7,8,10,11,28,31] \oplus S_{12}^7[0,7,16,23,24]\oplus \nonumber \\&=S_1^{7.25}[7,16,23,24]\oplus S_6^{7.25}[0,1,2,6,9,11,19,21,22,26,27,30,31] \oplus \nonumber \\&S_{11}^{7.25}[7,8,10,11,28,31] \oplus S_{12}^{7.25}[0,7,8,16,23]\nonumber \\&\,\hbox {that holds with probability}\,1. \end{aligned}$$
(iii) For the bits of group $\underline{Diagonal\,III}$, we get the linear approximations using Eqs. (10), (11) and (12) as follows:
    $$\begin{aligned} \begin{aligned} S_2^7[6,7,11,12,16,18,19]=S_2^{7.25}[7,12,16,19]\oplus S_7^{7.25}[7,12,15,16,19]\\ \,\hbox { holds with probability}\,\frac{1}{2}\left( 1+\frac{1}{2^4}\right) . \end{aligned} \end{aligned}$$
    $$\begin{aligned} \begin{aligned}S_{13}^7[6,8,18,19,23,25,26]=&S_{13}^{7.25}[6,8,18,19,23,25,26]\\&\oplus S_{13}^{7.25}[2,3,7,9,10,22,24]\\&\,\hbox { holds with probability}\,1.\end{aligned} \end{aligned}$$
    $$\begin{aligned} \begin{aligned}S_7^7[14,17,18,19]=S_7^{7.25}[14,17,18,19]\,\hbox {holds with probability}\,1.\end{aligned} \end{aligned}$$
    $$\begin{aligned} \begin{aligned} S_8^7[0,12,16,23]=S_8^{7.25}[0,12,16,23]\,\hbox {holds with probability}\,1.\end{aligned} \end{aligned}$$
  By combining these above approximations, we get the following linear approximation
  16 $$\begin{aligned}{}&S_2^7[6,7,11,12,16,18,19]\oplus S_7^7[14,17,18,19]\oplus \nonumber \\&S_8^7[0,12,16,23]\oplus S_{13}^7[6,8,18,19,23,25,26]\nonumber \\&\quad =S_2^{7.25}[6,7,8,12,16,18,23,25,26]\oplus S_7^{7.25}[7,12,14,15,16,17,18]\oplus \nonumber \\&S_8^{7.25}[0,12,16,23]\oplus S_{13}^{7.25}[2,3,7,9,10,22,24]\nonumber \\&\quad \hbox {that holds with probability}\,\frac{1}{2}\left( 1+\frac{1}{2^4}\right) . \end{aligned}$$
(iv) For the bits of group $\underline{Diagonal\,IV}$, we get the linear approximations using Eqs. (10), (11) and (12) as follows:
    $$\begin{aligned} \begin{aligned}S_3^7[0,7,8,11,12,16,22,23,28]=&S_3^{7.25}[0,8,12,16,23,28]\\&\oplus S_4^{7.25}[0,8,12,15,16,23,27,28]\\&\quad \hbox {holds with probability}\,\frac{1}{2}\left( 1+\frac{1}{2^5}\right) .\end{aligned} \end{aligned}$$
    $$\begin{aligned} \begin{aligned}{}&S_{14}^7[3,6,8,11,12,14,19,20,26,27] =S_3^{7.25}[3,6,8,11,12,14,19,20,26,27]\oplus \\&S_{14}^{7.25}[3,4,10,11,19,22,24,27,28,30]\\&\quad \hbox {holds with probability}\,1.\end{aligned} \end{aligned}$$
    $$\begin{aligned} \begin{aligned}S_4^7[7,19,23,30,31]=S_4^{7.25}[7,19,23,30,31]\,\hbox {holds with probability}\,1.\end{aligned} \end{aligned}$$
    $$\begin{aligned} \begin{aligned}S_9^7[3,4,6,8,15,16,19,20,26,31]&=S_9^{7.25}[3,4,6,8,15,16,19,20,26,31]\\&\quad \hbox {holds with probability}\,1. \end{aligned} \end{aligned}$$
  By combining these above approximations, we get the following linear approximation
  17 $$\begin{aligned}{}&S_3^7[0,7,8,11,12,16,22,23,28]\oplus S_4^7[7,19,23,30,31]\oplus \nonumber \\&S_9^7[3,4,6,8,15,16,19,20,26,31]\oplus S_{14}^7[3,6,8,11,12,14,19,20,26,27]\nonumber \\&\quad =S_3^{7.25}[0,3,6,11,14,16,19,20,23,26,27,28]\oplus S_4^{7.25}[0,7,8,12,15,16,19,27,28,30,31]\oplus \nonumber \\&S_9^{7.25}[3,4,6,8,15,16,19,20,26,31]\oplus S_{14}^{7.25}[3,4,10,11,19,22,24,27,28,30]\nonumber \\&\quad \hbox {that holds with probability}\,\frac{1}{2}\left( 1+\frac{1}{2^5}\right) . \end{aligned}$$

Therefore, by combining the above approximations of Result 14, Result 15, Result 16, Result 17 and using Pilling-up Lemma^29^, we have the required result. Q.E.D.

#### Lemma 6

The following path of linear approximation from the 3.5 rounds to the 7.25 rounds of ChaCha

18 $$\begin{aligned} S_8^{3.5}[0]&= S_0^{7.25}[0,4,7,8,11,12,19,20,24,27,28,30,31]\oplus \nonumber \\&S_5^{7.25}[0,6,8,10,11,13,15,16,22,23,27]\oplus \nonumber \\&S_{10}^{7.25}[0,2,3,4,8,14,19,20,23,26,27,31] \oplus \nonumber \\&S_{15}^{7.5}[0,3,4,8,11,12,14,16,20,23,27,28] \oplus \nonumber \\&S_1^{7.25}[7,16,23,24]\oplus S_6^{7.25}[0,1,2,6,9,11,19,21,22,26,27,30,31] \oplus \nonumber \\&S_{11}^{7.25}[7,8,10,11,28,31] \oplus S_{12}^{7.25}[0,7,8,16,23]\oplus \nonumber \\&S_2^{7.25}[6,7,8,12,16,18,23,25,26]\oplus S_7^{7.25}[7,12,14,15,16,17,18]\nonumber \\&S_8^{7.25}[0,12,16,23]\oplus S_{13}^{7.25}[2,3,7,9,10,22,24]\oplus \nonumber \\&S_3^{7.25}[0,3,6,11,14,16,19,20,23,26,27,28]\oplus S_4^{7.25}[0,7,8,12,15,16,19,27,28,30,31]\oplus \nonumber \\&S_9^{7.25}[3,4,6,8,15,16,19,20,26,31]\oplus S_{14}^{7.25}[3,4,10,11,19,22,24,27,28,30], \end{aligned}$$

holds with probability $\frac{1}{2}(1+\frac{1}{2^{58}}).$

#### Proof

The proof follows from Lemma 5 and Result 5. Q.E.D.

We prove the linear approximation from the 7th round to the 7.25th round of the permutation in Result 5. The corresponding computational linear correlation is provided in the following Experiment 5.

#### Experiment 5

The linear approximation of the Result 5 holds computationally with linear correlation $\epsilon _{l8}=0.000245\approx 2^{-12}$. This correlation is confirmed using $2^{40}$ random samples.

#### Complexity

Here we discuss the complexity of differential-linear distinguisher for the 7.25 rounds ChaCha permutation. The differential correlation $(\epsilon _d)$ for 3.5 rounds of the cipher is $\epsilon _d=2^{-28.65}.$ Now, using the computational linear correlations from Experiment 1, Experiment 2, Experiment 3, Experiment 4, and Experiment 5, we get the differential-linear correlation $\epsilon _d(\epsilon _l\epsilon _{l8})\approx 2^{-113}$ for the 7.25-round of the cipher. Therefore, the data and time complexity for the 7.25-round differential-linear distinguisher is $2^{231}$ as the attack has to repeat $2^5$ times on average.

## Conclusion

In this paper, we present a new 3.5-round of single-bit differential distinguisher. Then using this differential distinguisher, we find a new path of linear approximation that yields a $2^7$ times better differential-linear distinguisher than^27^ for 7-round ChaCha permutation. Also, we extend this 7-round distinguisher for the first time to a 7.25-round differential-linear distinguisher with data and time complexity $2^{231}.$ We address the issue of massive data generation for the attack that was not mentioned in the previous distinguishing attack on ChaCha. The existing multibit distinguishing attacks on 7-round ChaCha^24,27^ do not work for the cipher. We mention the reason that these attacks are only valid for the ChaCha permutation.

## Data Availability

One can generate data using our source codes. The source codes of our results are publicly available at https://www.dropbox.com/scl/fo/d2prs6kqk3n43p7fn9w10/h?dl=0 &rlkey=m0k3z4l3mjjng11qzoq6grirp.
